# Dietary Diversity and its Association with Nutritional Status, Cardiometabolic Risk Factors and Food Choices of Adults at Risk for Type 2 Diabetes Mellitus in Cape Town, South Africa

**DOI:** 10.3390/nu14153191

**Published:** 2022-08-04

**Authors:** Samukelisiwe S. Madlala, Jillian Hill, Ernesta Kunneke, Andre P. Kengne, Nasheeta Peer, Mieke Faber

**Affiliations:** 1 Non-Communicable Diseases Research Unit, South African Medical Research Council, Cape Town 7505, South Africa; 2 School of Public Health, Faculty of Community and Health Sciences, University of the Western Cape, Bellville 7535, South Africa; 3 Department of Dietetics and Nutrition, University of the Western Cape, Bellville 7535, South Africa; 4 Department of Medicine, University of Cape Town, Cape Town 7935, South Africa

**Keywords:** dietary diversity, nutritional status, food choices, adults, diabetes risk, cardiometabolic, South Africa

## Abstract

In South Africa, the nutrition transition has led to unhealthy diets lacking variety, contributing to the rise in overweight, obesity and diet-related noncommunicable diseases. Using baseline screening data of the South African Diabetes Prevention Programme (SA-DPP) study, this study aims to determine the relationship of dietary diversity (DD) with nutritional status, cardiometabolic risk factors and food choices of adults at risk of type 2 diabetes in resource-poor communities around Cape Town. Data of 693 adults, 25–65 years old were analysed. This included socio-demographic information, anthropometric measurements, biochemical assessments, food groups consumed the previous day and consumption frequency of certain foods to reflect food choices. The Minimum Dietary Diversity for Women (MDD-W) indicator was calculated; 70.4% of participants had low DD (<5 food groups). Low DD was associated with elevated serum triglycerides [AOR: 1.49, 95% CI (1.03, 2.15) *p* = 0.036]. The DD score was positively correlated (although weak) with the unhealthy food score (r = 0.191, *p* = 0.050) and sugary food score (r = 0.139, *p* < 0.01). Study participants at risk of diabetes consumed a diet low in DD; however, DD was not associated with nutritional status or cardiometabolic risk factors except for serum triglycerides.

## 1. Introduction

Recent estimates show that seven out of ten leading causes of death worldwide are non-communicable diseases (NCDs), with type 2 diabetes mellitus (hereafter referred to as diabetes) being a key contributor to global mortality [1]. The global burden of diabetes is substantial with 537 million people between 20 and 79 years having diabetes [2]. In Africa, an estimated 24 million people had diabetes in 2017 [2]. South Africa has the largest population of people with diabetes in sub-Saharan Africa, with approximately 4.2 million people between the ages of 20 and 79 years with diabetes [2]. Diabetes is the sixth leading cause of death in South Africa [3]; accounting for 7% of NCD-related mortality [4]. Diabetes frequently clusters with overweight/obesity and dyslipidaemia, and all are common risk factors for cardiovascular diseases (CVDs) [5]. The increased incidence of diabetes and other NCDs in South Africa is influenced by urbanisation leading to lifestyle changes such as the uptake of unhealthy diets and physical inactivity. This contributes to the development of overweight/obesity and subsequent diabetes [6].

Quality diets are associated with adequate intake of micro- and macronutrients, healthy dietary patterns, and reduced risk of diet-related diseases. Diet quality consists of four components namely adequacy, moderation, balance and variety [7]. Consuming a variety of nutritious foods is recommended globally through food-based dietary guidelines (FBDGs) [8]. While validated dietary diversity (DD) indicators can be used as a proxy for micronutrient adequacy [9], DD scores can also be used to reflect the variety of nutritious food in the diet. Whilst diverse diets are said to prevent chronic diseases [10], research on DD measures and health outcomes has yielded conflicting results. Some studies suggest that DD is associated with reduced risk for the development of NCDs [11,12,13], while others showed DD to be associated with overweight and obesity in adults and the occurrence of NCDs [14,15]. According to an American Heart Association science advisory, greater DD is not associated with better diet quality and healthy weight status [16]. Some studies showed DD to be associated with higher intakes of processed foods, refined grains and sugar-sweetened beverages (SSB) and lower intakes of unrefined foods, fish, fruits and vegetables [14,16]. In contrast, a study in young female adults showed that high DD was associated with consumption of healthy foods and lower consumption of high fat foods and refined grains [17]. The lack of clarity on the definition for DD and how it is measured across various settings could have contributed to the inconsistent findings across studies. Moreover, there is a wide variety of DD measures which differ in terms of food groups selected, food items counted and reference periods [16,18]. There is a need to develop better indicators that measure healthy, unhealthy and imbalanced DD. Such indicators would assist in addressing the burden of malnutrition especially in low-income populations [18].

South Africa is classified as an upper middle-income country, and the ongoing nutrition transition has led to a significant rise in the consumption of processed foods, soft drinks and fast foods [19]; simultaneously, the adult population in general consume a diet low in variety [20,21]. The lack of dietary diversity among the population may be due to various factors such as low incomes and unemployment, which may limit vulnerable population groups having access to a variety of healthy foods [21]. The widespread intake of cheap unhealthy foods contributes to high overweight and obesity rates and subsequently to NCDs which are the main disease burden in South Africa [20,22]. Within this context, the aim of this study was to determine DD and its association with nutritional status (anthropometric status), cardiometabolic risk factors (plasma glucose levels and serum lipids) and food choices (intake of unhealthy foods and food practices) in adults at risk of type 2 diabetes.

## 2. Materials and Methods

### 2.1. Study Design and Population

This is a cross-sectional study using baseline screening data collected between August 2017 and July 2019 among 25–65-year-old Black and Mixed ancestry adults enrolled in the South African Diabetes Prevention Programme (SA-DPP). The SA-DPP is a cluster randomised control trial with the aim to prevent the progression of pre-diabetes to diabetes in resource poor communities in the Cape Town metropolis of the Western Cape province in South Africa. The methods of the SA-DPP have been described elsewhere [23]. Briefly, Geographical Information Systems mapping was used to randomly select households within 16 suburbs/townships to identify potential participants. When the random sampling was proving to be unsuccessful, self-selection sampling was used to recruit participants in the townships [23]. The townships and suburbs were chosen based on previous studies that showed that those who are resource poor and at high risk of diabetes are located in these areas [24,25]. The average household monthly income for Cape Town residents is R3500 ($230.94) [26]. Poor urban households in Cape Town spend one-third of their total household income on food. In 2021 the unemployment rate in the Western Cape province was reported as 21.6% [27]. In Cape Town, the Black population has the highest unemployment rate (31.0%) followed by the Mixed-ancestry population (23.5%) [28].

### 2.2. Ethics

The baseline survey of the SA-DPP was approved by the ethics committee of the South African Medical Research Council (approval no. EC018-7/2015). The present study is part of a PhD study, which was approved by the University of the Western Cape Biomedical Research Ethics Committee (approval no. BM20/1/1).

### 2.3. Diabetes Risk Screening

Diabetes risk screening was done in two phases. Phase one involved risk screening of community members, whereby trained fieldworkers took anthropometric and blood pressure (BP) measurements and administered a short questionnaire (age, gender, ethnicity, previous diagnosis of diabetes and medical family history). In this phase, risk of diabetes was determined using the African Diabetes Risk Score (ADRS), which is based on age, body mass index (BMI), hypertension and waist circumference (WC) [23]. Participants identified as being at high risk were invited to participate in the second phase which was conducted at the research clinic at the Non-communicable Diseases Research Unit of the South African Medical Research Council. 

The second phase involved a more comprehensive assessment to identify those at high risk of developing diabetes, including oral glucose tolerance tests (OGTTs). Anthropometric and BP measurements were repeated by trained fieldworkers. Blood samples for glucose and lipids were collected by a qualified nurse from each participant after a 10 h overnight fast. Participants completed an interviewer-administered questionnaire that included socio-demographic information, personal and family medical history, dietary history, alcohol and tobacco use. Eligible participants had to be 25–65 years old, fluent in English and/or Afrikaans or IsiXhosa, able to give informed consent and willing to participate in the intervention trial. Individuals previously diagnosed with diabetes, bedridden, pregnant/breastfeeding and those receiving either cancer and/or tuberculosis treatment (current or during the past 3 months) were excluded.

### 2.4. Socio-Demographic and Behavioural Risk Factors

Socio-demographic data included participant age, gender, ethnicity, education level, employment status, type of housing and household income. The participants were asked about their use of tobacco and alcohol consumption (WHO STEPwise surveillance questionnaire) [29]. 

### 2.5. Anthropometric Measurements

Anthropometric measurements were taken twice by trained fieldworkers according to standard procedure [30]. Anthropometric measurements included weight (kg), height (cm), WC (cm) and hip circumference (HC) (cm). Participants were weighed using the UC-321 Precision health scale wearing light clothing and without shoes. Weight was recorded in kilograms to the nearest 0.1 kg. Standing height was measured using a portable SECA Leicester height measure. Participants were requested to stand up straight, feet flat and head in the Frankfort horizontal plane position. The WC measurement was taken midway between the lower border of lowest rib and upper border of iliac crest/pelvic bone using a SECA 201 flexible measuring tape. The HC measurement was measured around the widest portion of the buttocks, with the tape measure parallel to the floor. Height, WC and HC measurements were recorded to the nearest 0.1 cm. 

Weight and height measurements were used to calculate BMI [weight (kg)/height (m)^2^]. This was categorised as either underweight (BMI < 18.5 kg/m^2^), normal weight (BMI 18.5–24.9 kg/m^2^), overweight (BMI 25.0–29.9 kg/m^2^) or obese (BMI ≥ 30 kg/m^2^) [31]. Waist-to-hip ratio (WHR) was calculated by dividing the WC by the HC. According to the World Health Organisation (WHO) a normal WHR is 0.90 cm or less for males and 0.85 cm or less for females [32]. 

### 2.6. Biomedical Indicators

Fasting blood samples were taken for glucose and lipid levels, followed by a standard OGTT using 75 g glucose load diluted in 250 mL of water administered to participants and blood sample taken after 120 min. Blood samples were analysed at the PathCare laboratories for 2-h OGTT, glycated haemoglobin (HbA1c), serum total cholesterol (TC), triglycerides (TG), high density lipoprotein cholesterol (HDL-C) and low density lipoprotein cholesterol (LDL-C). The enzymatic hexokinase method was used to determine plasma glucose levels (Beckman AU, Beckman Coulter, Cape Town, South Africa). The HbA1c was measured using high performance liquid chromatography (Biorad Variant Turbo, BioRad, Johannesburg, South Africa). Enzymatic colorimetric methods were used to measure HDL-C and TG. The LDL-C was calculated using the Friedewalds formula.

Glycaemic status was defined according to the 1998 WHO definition [33]. Normoglycemia was defined as fasting plasma glucose (FPG) ≤ 6 mmol/L and 2 h glucose load < 7.8 mmol/L; and high-risk for developing type 2 diabetes (prediabetes) as FPG 6.1–7 mmol/L and 2-h glucose load ≥ 7.8–11.1 mmol/L. Diabetes was defined as FPG > 7 mmol/L and/or 2-h glucose load > 11.1 mmol/L. Abnormal blood lipid profile was defined as TC ≥ 5 mmol/L, HDL-C < 1.2 mmol/L, LDL-C ≥ 3 mmol/L and TG > 1.5 mmol/L [34]. 

### 2.7. Food Groups Consumed and Dietary Diversity 

Dietary diversity of study participants was assessed using the Minimum Dietary Diversity for Women (MDD-W) [9]. The MDD-W is a validated population-level indicator for women of reproductive age and reflects the micronutrient adequacy component of diet quality [9]. There is no DD measure available that has been validated specially for men or older women, and the MDD-W indicator has been used as measure of DD in studies with both men and women of all ages [35,36,37]. The MDD-W as measure of DD was therefore used in this study, regardless of age and gender. Participants were asked to recall all foods and drinks consumed the previous day and night, which were then allocated to pre-defined food groups. Dietary diversity was based on the 10 food groups of the MDD-W. The 10 food groups were namely: (1) grains, roots and tubers, (2) pulses (beans, peas and lentils), (3) nuts and seeds, (4) dairy, (5) meat, poultry and fish, (6) eggs, (7) dark green leafy vegetables, (8) other vitamin A rich fruits and vegetables, (9) other vegetables and (10) other fruits [9]. For each food group, a score of 1 was given if at least one food item within the food group was consumed in the preceding 24 h, and a score of 0 was given if no food item within the food group was consumed. The scores of the 10 food groups were summed to obtain the DD score. Participants with a DD score < 5 were classified as having low DD and those with DD scores ≥ 5 were classified as having adequate DD [9]. In addition, participants were categorised into quintiles based on the DD score, and food groups consumed by at least 50% of participants within each quintile were determined. An unhealthy food subscale score was calculated by summing the scores of five unhealthy food groups consumed in the preceding 24 h. These food groups were: (1) oils and fats, (2) sweets, (3) savoury and fried snacks, (4) SSB and (5) biscuits, and cakes and confectionery. The unhealthy food score could therefore range from 0–5. 

### 2.8. Food Choices and Practices

Frequency of intake over the past seven days was recorded for unhealthy foods such as processed meat, food covered with pastry or crumbs, food deep-fried in oil/fat, butter, ghee, fat, margarine or oil, mayonnaise or salad dressing, cookies, sweets, snacks, salty foods, sugar-sweetened cold drink, food from fast food outlets excluding beverages and fried food bought from street vendors. Frequency of intake of fruit juice, fruits and vegetables over the past seven days was also recorded. Frequency of consumption was recorded as none, 1–3 times, 4–6 days and every day. A sugary food score was calculated based on the frequency of consumption for three foods, namely cookies, sweets and SSB. For each of these three foods, frequency of consumption was scored as none = 0, 1–3 times = 2, 4–6 times = 5 and every day = 7. The scores for the three foods were summed to get a total sugary food score, which could range from 0–21. The score was then recategorised into food frequency categories; 0 = none, 1–6 = 1–3 times/week, and 7–21 = at least 4 times/week. 

The main reasons preventing daily intake of fruit and vegetables respectively were recorded. Participants reported food preferences concerning eating red meat with or without fat, eating chicken with or without the skin, adding salt to food, and the amount of margarine, butter or fat usually spread on bread, crackers or scones.

### 2.9. Statistical Analysis

Data were analysed using the statistical software package IBM SPSS for Windows version 27 (Armonk, New York, NY, USA). The Kolmogorov–Smirnov test and visual inspection of histograms, normal Q-Q plots and box plots were used to test for normality of the data distribution. Continuous variables are presented as means and standard deviations for normally distributed variables and as median and interquartile range for non-normally distributed variables. Categorical variables are presented as counts and percentages. Differences between groups were tested using the Mann Whitney U test for continuous variables that were not normally distributed, and the Pearson chi-square test for categorical variables using Bonferroni corrections. Since the data were not normally distributed, Spearman correlation analysis was done to determine the relationship of the DD score with the unhealthy food and sugary food scores, respectively. Binary and multinomial logistic regression analyses were used to calculate odds ratios (OR) and 95% confidence intervals (95% CI) for the associations between DD (low vs. adequate) as the independent variable, and dependent variables BMI (normal weight vs. overweight and obese), WHR (normal vs. high), glycaemic status (normoglycaemia vs. prediabetes vs. diabetes), TC (normal vs. elevated), HDL-C (normal vs. low), LDL-C (normal vs. elevated) and TG (normal vs. elevated). Adjusted OR (AOR) were calculated by adjusting for gender and ethnicity (model 1), and gender, ethnicity and age (model 2). All statistical tests were considered significant at *p* < 0.05.

## 3. Results

### 3.1. Socio-Demography and Behavioural Risk Factors

Baseline data were available for 700 participants, but seven participants were excluded due to incomplete/missing data. Data analysis was therefore based on 693 participants. The mean age of the study participants was 50.9 ± 9.1 years. The majority of the participants (*n* = 488, 70.4%) consumed a diet of low DD (fewer than 5 food groups) and 205 (29.6%) consumed a diet of adequate DD (at least 5 food groups). Table 1 shows the sociodemographic characteristics of the total study sample and for the two DD categories. Most participants were female (81.1%). Unemployment was high (43.7%), and the majority (71.6%) had low household incomes [≤R3200 (US $200.27)]. Most participants consumed alcohol (63.9%) and a quarter smoked tobacco. Participants with low DD vs. adequate DD had less formal schooling (<grade 12; 86.0% vs. 79.9%) were less likely to live in built formal unit/privately owned housing (32.2% vs. 42.9%) and had lower household income [≤R 3200 (US $200.27); 75.4% vs. 62.6%].

### 3.2. Dietary Diversity Food Groups

**Figure 1:**
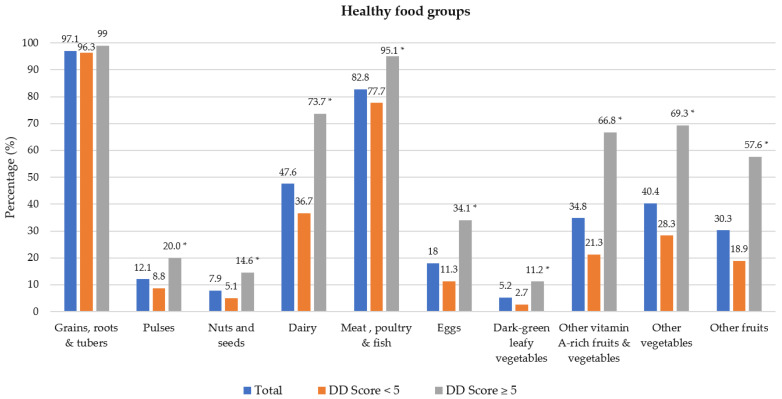
Percentage of participants who consumed healthy food groups the previous day by dietary diversity (DD) score categories. * Significant difference between DD score categories at *p* < 0.001 level, Chi-square test.

**Figure 2:**
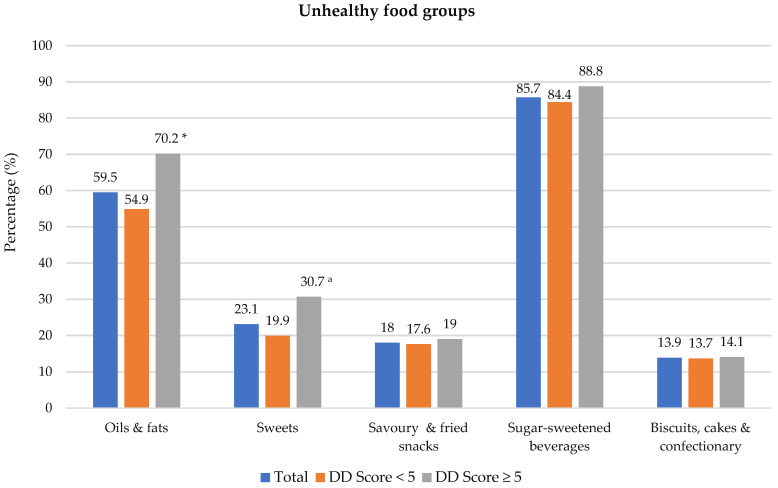
Percentage of participants who consumed unhealthy food groups the previous day dietary diversity (DD) score categories. * Significant difference between DD score categories at *p* < 0.001 level, Chi-square test. ^a^ Significant difference between DD score categories at *p* < 0.05 level, Chi-square test. Sugar-sweetened beverages include tea/coffee with sugar, cool drink, fruit juice, flavoured water and energy drink.

Food groups consumed the previous day are presented in Figure 1. Of the 10 healthy food groups, the most consumed food groups were grains/roots/tubers (97.1% of participants) and meat/poultry/fish (82.8%). The least consumed food groups were dark-green leafy vegetables (5.2%), nuts and seeds (7.9%) and pulses (12.1%). A significant difference was noted between the two DD categories for all food groups except for grains/root/tubers; a higher proportion of participants with DD score ≥ 5 consumed foods from the different food groups. Regarding unhealthy food groups, a higher percentage of participants with adequate DD in comparison to those with low DD consumed oils and fats (54.9% vs. 70.2%) and sweets (19.9% vs. 30.7%) during the recall period (Shown in Figure 2). Spearman correlation analysis showed a weak positive relationship between the DD score and the unhealthy foods score (r = 0.191, *p* = 0.050).

Table 2 shows the food groups that were predominantly consumed (by at least 50% of participants) within each DD score quintile. Grains/roots/tubers and meat/poultry/fish were the only two food groups that were consumed by at least 50% of participants in the two lowest DD quintiles. As DD increased, dairy became predominant, followed by other vitamin A-rich fruits and vegetables, and other vegetables. Other fruit and eggs were predominantly consumed in the highest DD quintile only.

### 3.3. Food Choices 

**Figure 3:**
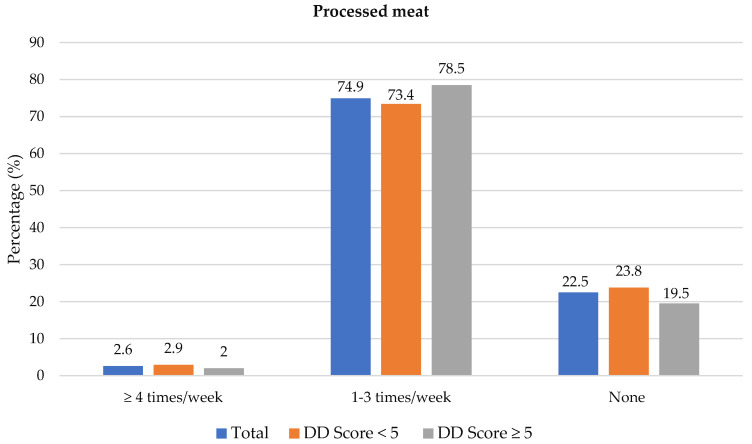

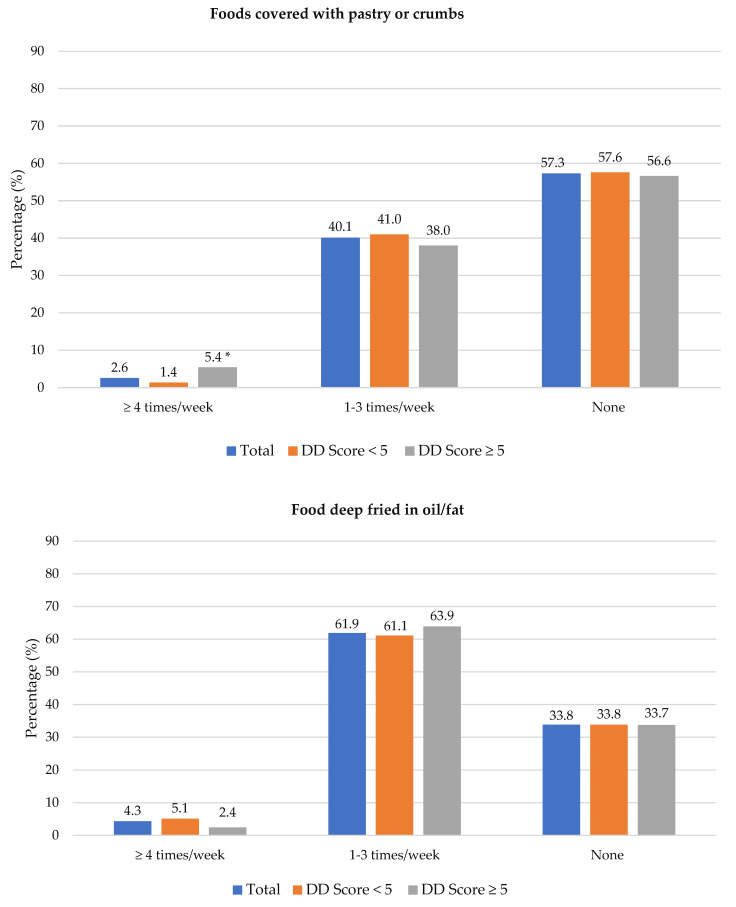

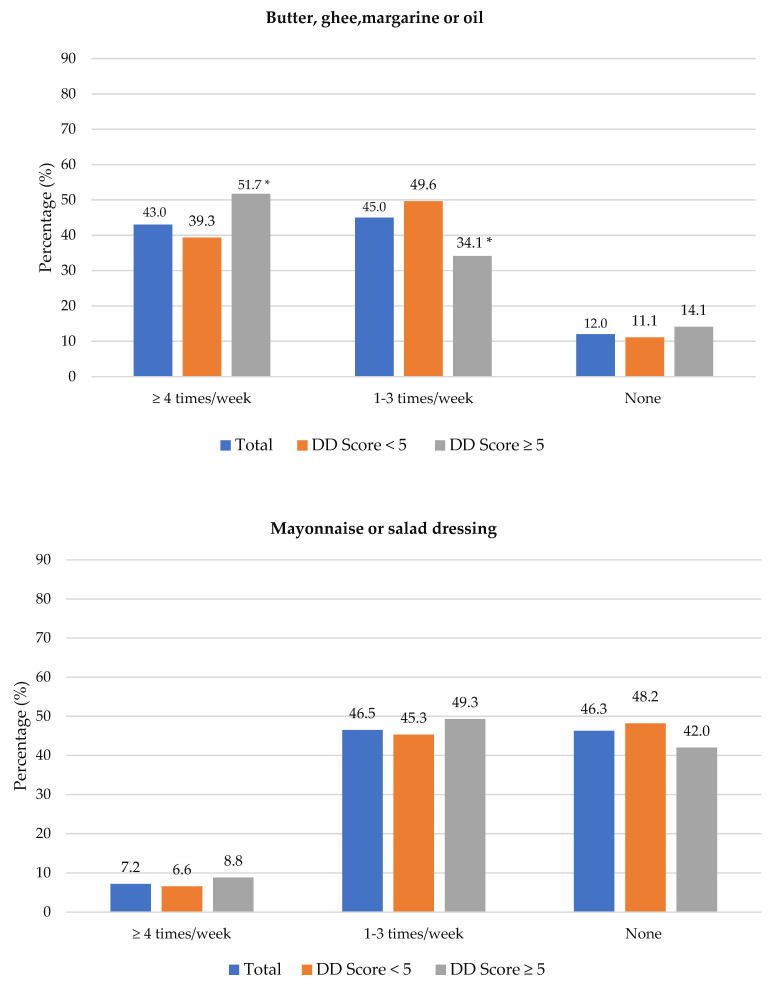

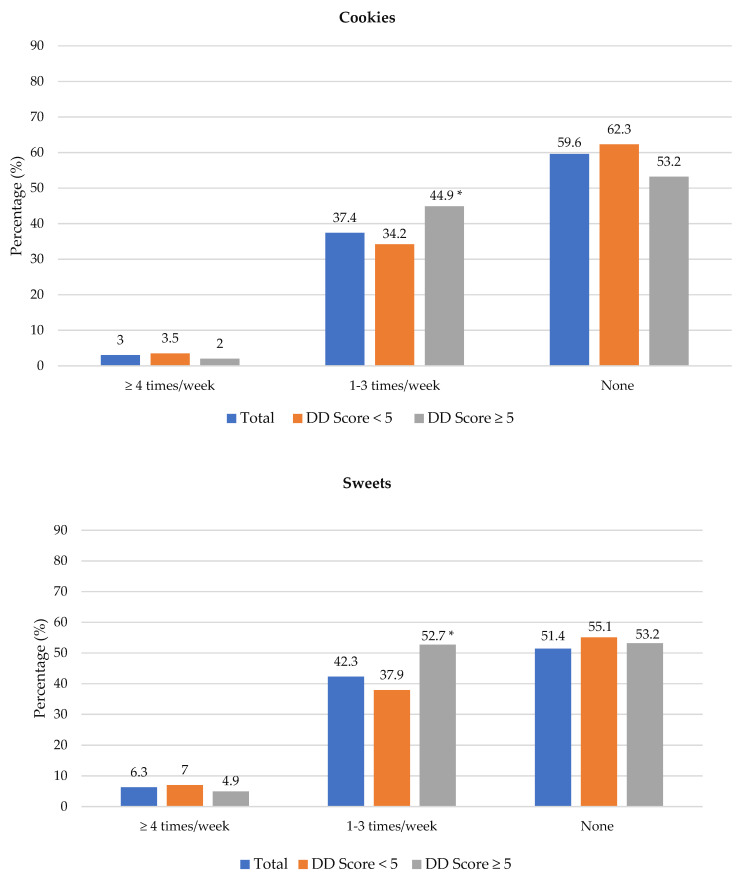

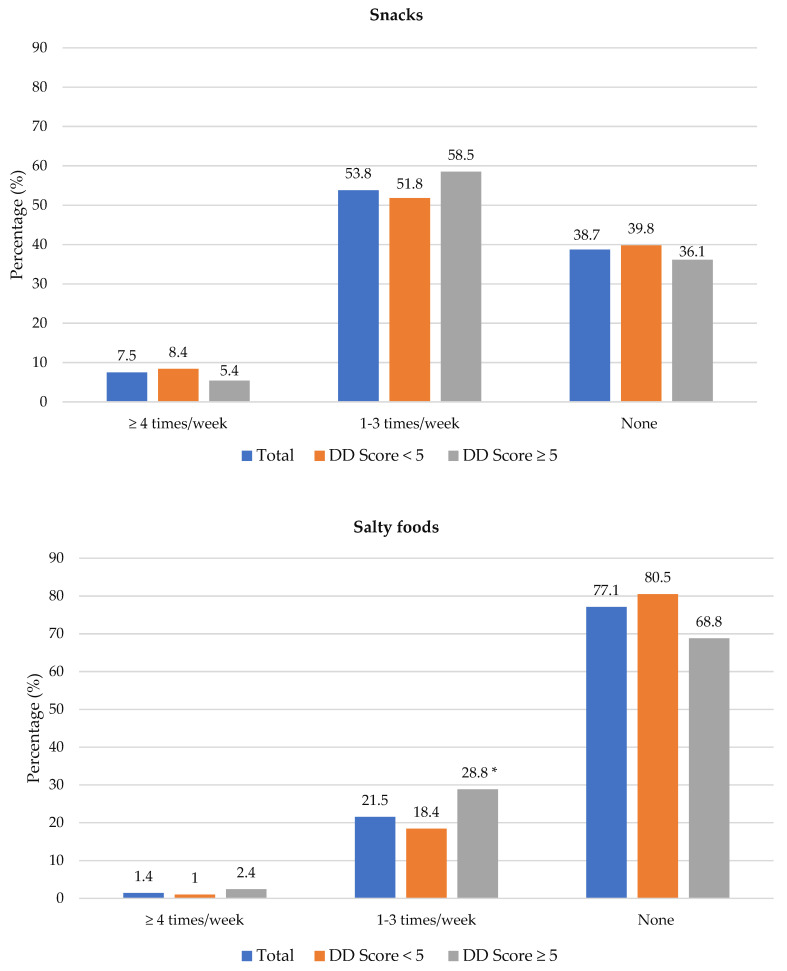

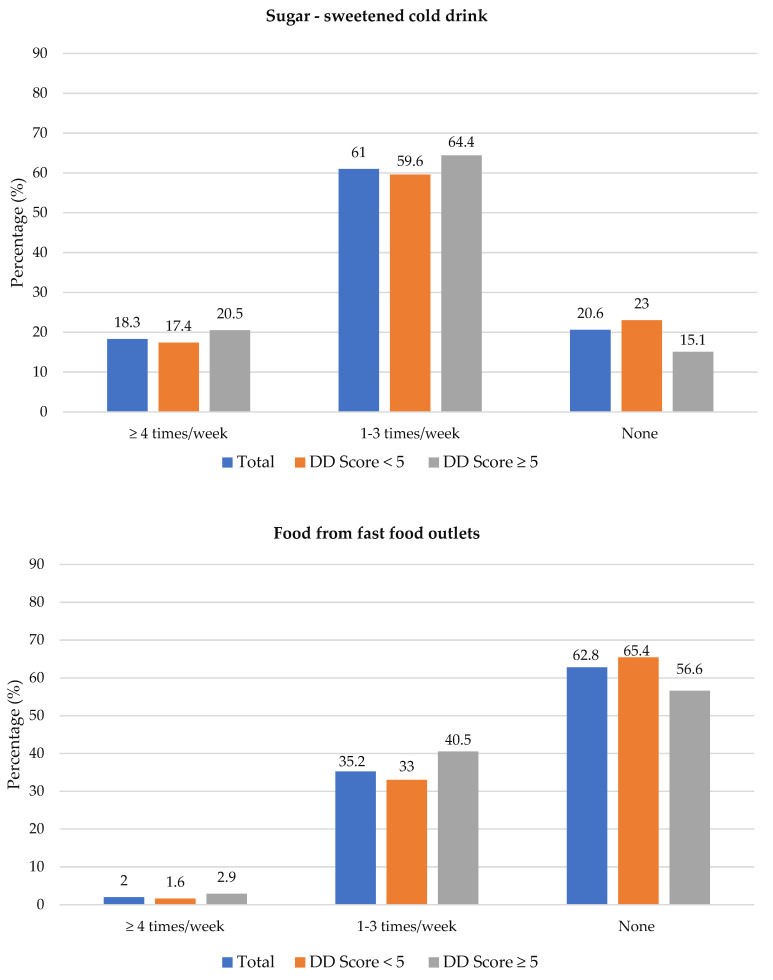

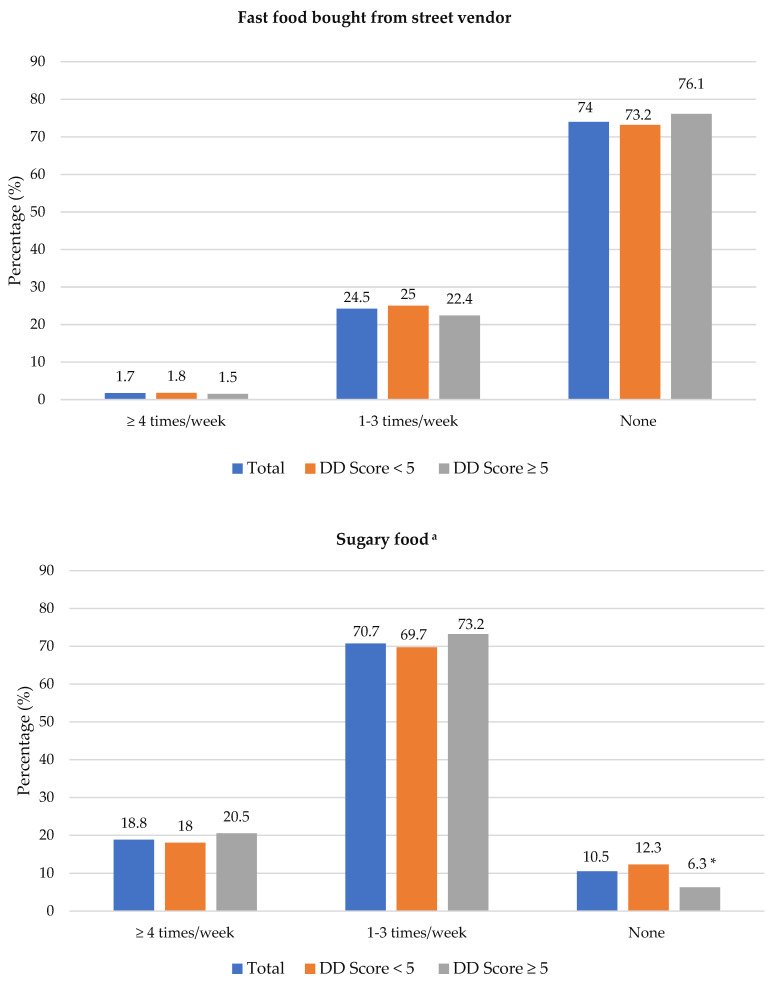
Frequency intake of selected unhealthy foods by dietary diversity (DD) score categories. * Significant difference between DD score categories at *p* < 0.05 level; ^a^ Based on a calculated sugary food score.

The frequency of consumption of selected foods was used to reflect food choices. Figure 3 shows the frequency of consumption of selected unhealthy foods. Participants with adequate DD more frequently consumed foods covered with pastry/crumbs, butter/ghee/margarine/oil (at least four times/week), cookies, sweets and salty foods (1–3 times/week) than participants with low DD. The Spearman correlation test showed a weak positive relationship between the DD score and the sugary food score (*r* = 0.139, *p* < 0.01). 

**Figure 4:**
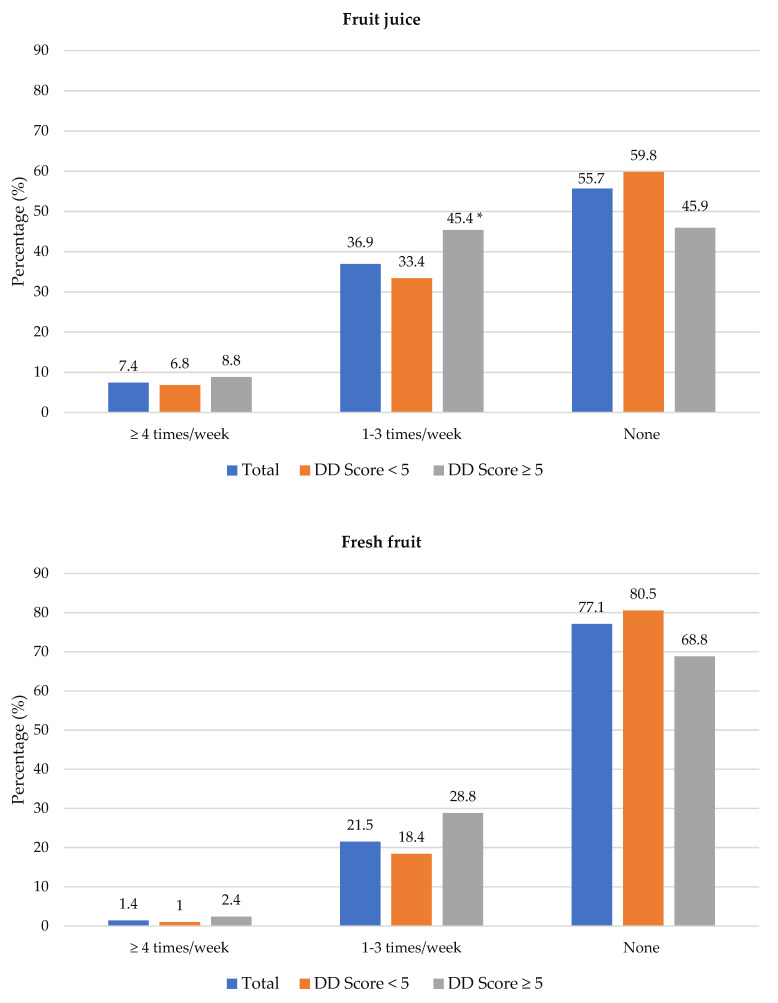

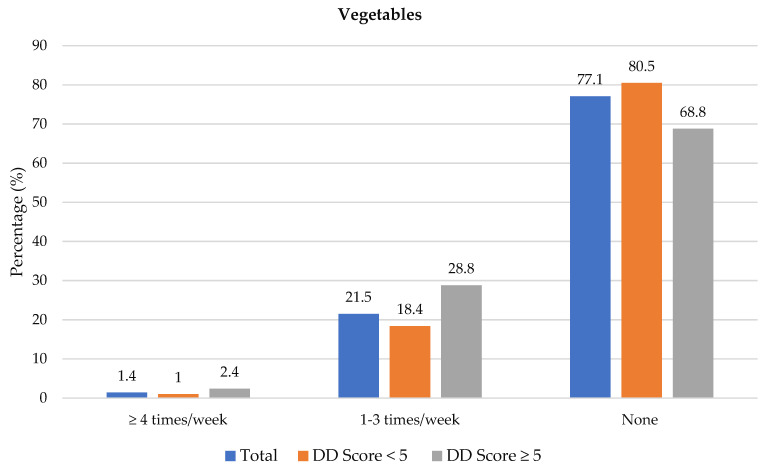
Frequency intake of fruit juice, fresh fruit and vegetables by dietary diversity (DD) score categories. * Significant difference between DD score categories at *p* < 0.05 level.

Figure 4 shows the frequency of consumption of fruit juice, and fresh and vegetables. Participants with adequate DD more frequently consumed fruit juice (1–3 times/week) than participants with low DD (45.4% vs. 33.4%). Fresh fruit was consumed at least 4 times/week by significantly more participants with adequate DD compared to those with low DD (41.5% vs. 25.6%). Overall, 23.5% (*n* = 163) participants consumed fresh fruit and 40.1% (*n* = 278) consumed vegetables daily (data not shown in table). Of the 693 participants, 42.6% did not eat fruit and 28.6% did not eat vegetables daily because of financial constraints. A higher percentage of participants with low DD compared to those with adequate DD reported financial constraints as barrier for daily intake of fruits (47.1% vs. 31.7%) and vegetables (32.0% vs. 20.5%) (Supplementary Table S1). 

None of the food practices differed significantly between the DD categories except for eating chicken with skin (low DD 60.0%, adequate DD 51.7%) (Supplementary Table S2).

### 3.4. Nutritional Status and Cardiometabolic Risk Factors

#### 3.4.1. Nutritional Status

The median BMI was 35.6 kg/m^2^ in the overall sample, and higher in women (36.9 kg/m^2^) than men (28.6 kg/m^2^); *p* < 0.05 (Table 3). Hip circumference measurements were not taken for the first 60 participants enrolled in the SA-DPP study; and therefore, WHR data are only available for 633 participants. The median WHR for males was 0.96 (0.93–1.00) and 0.91 (0.85–0.97) for females (Supplementary Table S3). Obesity, defined by BMI and WHR, at 77.1% and 75.3%, respectively, was high. 

#### 3.4.2. Cardiometabolic Risk Factors

The prevalence of diabetes, prediabetes and normoglycemia was 10.3%, 16.8% and 72.9% respectively (Table 3). The prevalence elevated TC, LDL-C and TG was 48.0%, 55.4% and 33.8%, respectively. Approximately 40.1% of participants had low HDL-C. Nutritional status and cardiometabolic risk factors per gender and ethnicity groups are presented in Supplementary Tables S3 and S4 respectively. 

Crude and multivariable adjusted ORs and 95% CIs for the association of low DD (score < 5) with nutritional status and cardiometabolic risk factors are presented in Table 4. Unadjusted binary and multivariable logistic regression showed no significant associations between DD and any of the nutritional status or cardiometabolic risk factors. After adjusting for gender and ethnicity, participants with low DD were 1.45 times more likely to have elevated TG concentrations [AOR: 1.45; 95% CI (1.03, 2.15); *p* = 0.048]; this association remained significant after additionally adjusting for age [AOR: 1.49, 95% CI (1.03, 2.15); *p* = 0.036].

## 4. Discussion

This study highlights that most study participants residing in resource-poor communities in Cape Town consumed a diet with low variety. Notably, participants with low DD had lower household incomes and less formal schooling. Participants with adequate DD, however, reported more frequent consumption of unhealthy foods such as foods covered in pastry or crumbs cookies, sweets and salty foods. The only cardiovascular risk factor associated with low DD was elevated TGs. 

In total, 70.4% of the study participants consumed fewer than 5 of the 10 healthy food groups the previous day, indicating that DD was generally low, which is consistent with the findings of other South African cross-sectional studies [20,21]. A national study showed that lower living standards (measured by degree of urbanisation, services and asset ownership) are associated with low DD [23]. Moreover, a South African study showed that healthier foods are generally less affordable than unhealthy foods [38]. Considering that in South Africa cost is the main factor influencing food choices when grocery shopping [20], together with the high unemployment rate of 35.3% [39] and high reliance on social grants (45.5% of households [40]), improving DD in resource-poor settings may be challenging. In an attempt to assist low-income households in spending less of their income on food, the South African government has VAT zero-rated 19 basic foodstuffs [41]. Although fruit and vegetables are VAT zero-rated, cost remains a barrier for frequent consumption [42].

Grains/roots/tubers and meat/poultry/fish were the two most consumed food groups and were the only predominantly consumed food groups in the two lowest DD score quintiles. Similar results were reported in a national study that determined DD in South Africans aged 16 years and older [21]. Our findings are further supported by a study that was done in formal and informal settings in Johannesburg, South Africa [43]. Comparatively, however, opposite results have been reported for other countries in Africa. For example, in a study in Nigeria, cereal and vegetables were reported as the most consumed food groups [44], while in Tanzania the most consumed food groups were cereals, vegetables, legumes, nuts and seeds, and fruit [45]. Due to rapid urbanisation and the nutrition transition, dietary intake in South Africa has shifted from traditional diets rich in fibre, lean meats, legumes, vegetables and fruits to more westernised diets that include energy dense, refined and ready prepared foods and less vegetables and fruits [46]. Food consumption data between 1994 and 2012 showed that in South Africa there was an increase in the consumption of meat, fats and oils, soft drinks, sweet and savoury snacks, while consumption of vegetables decreased [47]. 

Fruits and vegetables were predominantly consumed by participants in the two highest DD score quintiles only, and cost was the main barrier for daily consumption. Vegetables and fruit are amongst the least consumed food groups in South Africa [48], and per capita intake thereof is approximately 200 g [20], which is half the WHO recommendation of at least 400 g per day to protect against various NCDs [49]. Low intake of fruits accounted for two million global deaths and 65 million disability adjusted life years in 2017 [50]. Although daily consumption of fruits and vegetables is recommended, cost (affordability) has been cited as a major barrier for daily consumption not only in South Africa [42,51,52] but globally as well [42].

Adequate DD based on healthy foods was also associated with consumption of several unhealthy foods, and the DD score correlated positively with the sugary foods score. Similar findings were reported in an American cohort study which showed adequate DD to be associated with intakes of nutrient-dense foods such as fruits, vegetables and whole grains, as well as unhealthy foods such as processed meats, salty snacks and SSB [53]. In South Africa, unhealthy processed foods such as fried foods, fast food, salty snacks and processed meats are regularly consumed [54]. These foods are generally inexpensive and therefore more accessible and preferable to low-income households [20], and this has contributed to unhealthy diets, overweight/obesity and NCDs [46]. In 2012, Igumbor and colleagues argued that a development plan by the South African government to improve accessibility, affordability and acceptability of healthy foods and limiting the availability, discouraging the advertising and increasing the cost of unhealthy foods including soft drinks, packaged foods and snacks is warranted [55]. There are currently several legislations, regulations and policies in South Africa that aim to reduce the incidence of NCDs. For instance, the regulation on sodium reductions, a levy on salt substitutes and levy on SSBs [56], aim to decrease salt and sugar consumption as well as the prevalence of hypertension, heart disease, overweight and diabetes among the public.

Although our study found no association between DD and BMI status or WHR, previous studies have yielded contrasting results. Some studies have shown a positive association [57], another an inverse association [17] and a recent systematic review and meta-analysis reported that eight out of 16 studies found no association between DD and BMI status [58]. We found no associations between DD and the cardiometabolic risk factors, except for TG. Our finding that low DD was associated with elevated TG concentrations is similar to a cross-sectional study in Iranian adults [17]. There are many factors other than DD that may influence serum TG concentrations, such as consumption of sugary food and drinks, saturated and trans-fats, refined grains, high energy foods as well as alcohol [59] and overweight and obesity and tobacco use [60].

Our study included only participants with existing diabetes risk, most had low DD and almost all were either overweight or obese; this could have contributed to the lack of associations of DD with nutritional status and most of the cardiometabolic risk factors. The differences in our findings from other studies may also be attributable to the different study populations, dietary assessment methods and tools used to measure DD [18]. Dietary diversity indicators were developed mostly to be used as population-level proxy indicators and are based on a variety of healthy foods, but do not take less healthy foods into account. Dietary diversity indicators therefore do not reflect overall quality of the diet [18] and therefore their usefulness in NCD research may be limited. Although a diverse diet may be beneficial to health outcomes, studies show inconsistent results on the association of DD indicators with health outcomes. The recently developed Global Diet Quality Score (GDQS) is a more comprehensive population-level metric for both nutrient adequacy and diet-related NCD risk [61] may be a more suitable tool; however, the GDQS has not yet been validated in South Africa.

A strength of this study is that it includes a relatively large sample size to test for associations. The present study had several limitations that are important to note. The cross-sectional study design examined associations and therefore cannot determine causal relationships. Dietary diversity was based on the MDD-W score, which has not been validated for men and older women. Dietary data were based on self-report, and therefore, may be subject to error and recall bias. All participants included in the study were deemed at risk for diabetes on screening. The results can only be applied to adults at risk for diabetes living in resource-poor settings and cannot be generalised to the general population.

## 5. Conclusions

The findings of the study demonstrate that a high proportion of individuals from resource poor communities who were at risk for diabetes on screening consumed a diet with low variety. Overall, DD was not associated with nutritional status and cardiometabolic risk factors, except for the association of low DD with increased likelihood of elevated TGs. Adequate DD was associated with both healthy and unhealthy food choices, which further highlights the need to consider both healthy and unhealthy foods when constructing measures of dietary diversity.

## Figures and Tables

**Table 1:** Socio-demographic characteristics and behavioural risk factors of the study sample across dietary diversity categories.

Variable	Total (*n* = 693)	DD Score < 5 (*n* = 488)	DD Score ≥ 5 (*n* = 205)	*p* Value ^a^
Number of participants	693 (100)	488 (70.4)	205 (29.6)	
Age, years, *n* (%)				0.117
25–44 years	155 (22.4)	119 (24.4)	36 (17.6)	
45–54 years	256 (36.9)	179 (36.7)	77 (37.6)	
55–65 years	282 (40.7)	190 (38.9)	92 (44.9)	
Gender, *n* (%)				0.151
Male	131 (18.9)	99 (20.3)	32 (15.6)	
Female	562 (81.1)	389 (79.7)	173 (84.4)	
Ethnicity ^b^, *n* (%)				0.392
Mixed-Ancestry	281 (40.7)	193 (39.6)	88 (43.1)	
Black	410 (59.3)	294 (60. 4)	116 (56.9)	
Marital status ^b^, *n* (%)				0.495
Single	217 (31.5)	161 (33.2)	56 (27.5)	
Married	296 (43.0)	202 (41.6)	94 (46.1)	
Divorced	72 (10.4)	53 (10.9)	19 (9.3)	
Widowed	64 (9.3)	43 (8.9)	21 (10.3)	
Other ^c^	40 (5.8)	26 (5.4)	14 (6.9)	
Education level ^b^, *n* (%)				**0.038**
<Grade 12	580 (84.2)	419 (86.0)	161 (79.7)	
≥Grade 12	109 (15.8)	68 (14.0)	41 (20.3)	
Occupation, *n* (%)				0.445
Employed	237 (35.0)	161 (33.6)	76 (38.2)	
Unemployed ^d^	296 (43.7)	216 (45.1)	80 (40.2)	
Pensioner/Disability grant	145 (21.4)	102 (21.3)	43 (21.6)	
Type of housing, *n* (%)				**0.024**
Built formal unit/privately owned	244 (35.4)	157 (32.2)	87 (42.9) *	
Council/core house	273 (39.6)	199 (40.9)	74 (36.5)	
Informal shack/shelter/hostel/other	173 (25.1)	131 (26.9)	42 (20.7)	
Monthly household income, *n* (%)				**<0.001**
R0–R3200	494 (71.6)	367 (75.4)	127 (62.6) *	
R3201–R6400	117 (17.0)	77 (15.8)	40 (19.7)	
R6401–R51200	79 (11.4)	43 (8.8)	36 (17.7) *	
Alcohol consumption during last 12 months, *n* (%)				0.165
≥5 days per week	4 (0.6)	3 (0.6)	1 (0.5)	
1–4 days per week	59 (8.5)	49 (10.0)	10 (4.9)	
Seldom (≤3 days per month)	187 (27.0)	131 (26.8)	56 (27.3)	
None	443 (63.9)	305 (62.5)	138 (67.3)	
Smoking status, *n* (%)				0.391
Non-smoker	519 (74.9)	361 (74.0)	158 (77.1)	
Smoker	174 (25.1)	127 (26.0)	47 (22.9)	

Data presented as *n* (%). DD—dietary diversity. ^a^ Chi-square test used for categorical variables, ^b^ Missing data were observed for some participants *n* = 4; ^c^ Other includes living as married; ^d^ Unemployed includes students and homemakers. * Significant difference between DD score categories at *p* < 0.05 level. Bold *p* value-significant at *p* < 0.05 & *p* < 0.001 level.

**Table 2:** Food groups consumed by more than 50% of participants per dietary diversity score quintile for the total study sample.

Quintile 1 (1–2 Groups) (*n* = 128)	Quintile 2 (3 Food Groups) (*n* = 175)	Quintile 3 (4 Food Groups) (*n* = 185)	Quintile 4 (5 Food Groups) (*n* = 139)	Quintile 5 (≥ 6 Food Groups) (*n* = 66)
Grains/roots/tubers	Grains/roots/tubers	Grains/roots/tubers	Grains/roots/tubers	Grains/roots/tubers
Meat/poultry/fish	Meat/poultry/fish	Meat/poultry/fish	Meat/poultry/fish	Meat/poultry/fish
		Dairy	Dairy	Dairy
			Other vitamin A-rich fruits and vegetables *	Other vitamin A-rich fruits and vegetables
			Other vegetables	Other vegetables
				Other fruits
				Eggs

* Other than dark green leafy vegetables.

**Table 3:** Nutritional status and cardiometabolic risk factors for the total study sample and the two dietary diversity categories respectively.

Variable	Total (*n* = 693)	DD Score < 5 (*n* = 488)	DD Score ≥ 5 (*n* = 205)	*p* Value ^a^
Gender				
Male	131 (18.9)	99 (20.3)	32 (15.6)	0.151
Female	562 (81.1)	389 (79.7)	173 (84.4)	
Ethnicity				
Mixed ancestry	281 (40.7)	193 (39.6)	88 (43.1)	0.392
Black	410 (59.3)	294 (60.4)	116 (56.9)	
BMI	35.6 (30.5–40.5)	35.6 (30.6–40.9)	35.4 (30.4–39.5)	0.579
Normal weight (18.5–24.9 kg/m^2^)	29 (4.2)	19 (3.9)	10 (4.9)	0.827
Overweight (25.0–29.9 kg/m^2^)	128 (18.6)	91 (18.7))	37(18.2)	
Obese (≥30 kg/m^2^)	533 (77.2)	377 (77.4)	156 (76.8)	
Total	690 (100)	487 (100)	203 (100)	
WHR	0.91 (0.86–0.97)	0.93 (0.87–0.97)	0.91 (0.85–0.97)	0.192
Normal ^b^	156 (24.7)	100 (22.5)	56 (29.8)	0.053
High ^c^	476 (75.3)	344 (77.5)	132 (70.2)	
Total	681 (100)	444 (100)	188 (100)	
Glycaemic status	6.0 (5.0–7.4)	5.9 (4.9–7.3)	6.1 (5.1–7.8)	0.643
Normoglycemia (FPG ≤ 6 and 2-h glucose < 7.8 mmol/L)	496 (72.9)	358 (74.7)	138 (68.7)	0.211
Prediabetes (FPG 6.1– 7 mmol/L and 2-h glucose ≥ 7.8–11.1 mmol/L)	114 (16.8)	77 (16.1)	37 (18.4)	
Diabetes (FPG > 7 mmol/L and 2-h glucose > 11.1 mmol/L)	70 (10.3)	44 (9.2)	26 (12.9)	
Total	680 (100)	479 (100)	201 (100)	
TC	4.9 (4.3–5.7)	4.9 (4.2–5.8)	5.0 (4.3–5.6)	0.783
Normal (<5 mmol/L)	451 (66.2)	255 (53.1)	99 (49.3)	0.356
Elevated (≥5 mmol/L)	230 (38.0)	225 (46.9)	102 (50.7)	
Total	681 (100)	480 (100)	201 (100)	
HDL-C	1.2 (1.1–1.4)	1.2 (1.1–1.4)	1.2 (1.1–1.4)	0.645
Normal (≥1.2 mmol/L)	272 (40.1)	192 (40.2)	80 (39.8)	0.929
Low (<1.2 mmol/L)	407 (59.9)	286 (59.8)	121 (60.2)	
Total	679 (100)	478 (100)	201 (100)	
LDL-C	3.1 (2.5–3.8)	3.1 (2.5–3.8)	3.1 (2.5–3.7)	0.856
Normal (<3 mmol/L)	81 (29.1)	215 (45.0)	88 (43.8)	0.774
Elevated (≥3 mmol/L)	197 (70.1)	263 (55.0)	113 (56.2)	
Total	679 (100)	478 (100)	201 (100)	
TG	1.3 (0.9–1.7)	1.3 (0.9–1.7)	1.2 (0.9–1.5)	0.402
Normal (≤1.5 mmol/L)	451 (66.2)	307 (64.0)	144 (71.6)	0.053
Elevated (>1.5 mmol/L)	230 (33.8)	173 (36.0)	57 (28.4)	
Total	681 (100)	480 (100)	201 (100)	

Data presented as median (interquartile range: IQR) or *n* (%). DD—dietary diversity; BMI—body mass index; WHR—waist-to-hip ratio; FPG—fasting plasma glucose; HDL-C—high density lipoprotein cholesterol; LDL-C—low density lipoprotein cholesterol; TG—triglyceride. ^a^ Chi-square test used for categorical variables and Spearman correlation for continuous variables, ^b^ Normal WHR: males ≤ 0.90 cm and females ≤ 0.85 cm, ^c^ High WHR: males > 0.90 cm and females > 0.85 cm.

**Table 4:** Odds ratios (95% confidence interval) of associations between low dietary diversity and nutritional status and cardiometabolic risk factors.

Variable	Crude Model OR (95% CI)	*p* Value	Model 1 AOR (95% CI)	*p* Value	Model 2 AOR (95% CI)	*p* Value
BMI						
Normal weight (18.5–24.9 kg/m^2^)	1		1		1	
Overweight and obese (≥25.0 kg/m^2^)	1.27 (0.58, 2.78)	0.550	1.58 (0.69, 3.62)	0.280	1.24 (0.53, 2.94)	0.619
WHR						
Normal ^a^	1		1		1	
High ^b^	1.46 (0.99, 2.14)	0.054	1.49 (0.99, 2.21)	0.052	1.45 (0.97, 2.16)	0.071
Glycaemic status						
Normoglycemia (FPG ≤ 6 and 2-h glucose < 7.8 mmol/L)	1		1		1	
Prediabetes (FPG 6.1–7 and2-h glucose ≥ 7.8–11.1 mmol/L)	0.80 (0.52, 1.24)	0.325	0.80 (0.52, 1.23)	0.337	0.82 (0.52, 1.31)	0.416
Diabetes (FPG > 7 and 2-h glucose > 11.1 mmol/L)	0.65 (0.39, 1.10)	0.109	0.63 (0.37, 1.07)	0.088	0.59 (0.34, 1.03)	0.062
TC						
Normal (<5 mmol/L)	1		1		1	
Elevated (≥5 mmol/L)	0.86 (0.62, 1.19)	0.357	0.87 (0.62, 1.22)	0.425	0.94 (0.66, 1.33)	0.715
HDL-C						
Normal (≥1.2 mmol/L)	1		1		1	
Low (<1.2 mmol/L)	0.99 (0.70, 1.38)	0.929	1.03 (0.73, 1.44)	0.882	1.09 (0.78, 1.55)	0.601
LDL-C						
Normal (<3 mmol/L)	1		1		1	
Elevated (≥3 mmol/L)	0.95 (0.68, 1.33)	0.774	0.99 (0.69, 1.39)	0.937	1.06 (0.74, 1.50)	0.760
TG						
Normal (≤1.5 mmol/L)	1		1		1	
Elevated (>1.5 mmol/L)	1.42 (0.99, 2.04)	0.054	1.45 (1.00, 2.09)	**0.048**	1.49 (1.03, 2.15)	**0.036**

OR—odds ratio, 95% CI—95% confidence interval, 1—reference. Model 1: adjusted for gender and ethnicity, Model 2: adjusted for gender, ethnicity and age. DD—dietary diversity, BMI—body mass index; WHR—waist-to-hip ratio; FPG—fasting plasma glucose; HDL-C—high density lipoprotein cholesterol; LDL-C—low density lipoprotein cholesterol; TG—triglyceride. ^a^ Normal WHR: males ≤ 0.90 cm and females ≤ 0.85 cm, ^b^ High WHR: males > 0.90 cm and females > 0.85 cm. Bold *p* value-significant at *p* < 0.05 level.

## Data Availability

Data used for this study are from baseline evaluation of the ongoing SA-DPP and are not available for sharing until trial completion.

## Supplementary Materials

The following supporting information can be downloaded at: https://www.mdpi.com/article/10.3390/nu14153191/s1, Table S1: Main reasons preventing eating fruits and vegetables every day per dietary diversity score category. Table S2: Food preference fat on meat and poultry, salt in food and use of margarine, butter & fat as spread according to ethnicity according to dietary diversity score category. Table S3: Nutritional status and cardiometabolic risk factors of participants according to gender and dietary diversity categories. Table S4: Nutritional status and cardiometabolic risk factors of participants according to ethnicity and dietary diversity categories.
