# Supplementary material for: Dietary Diversity and its Association with Nutritional Status, Cardiometabolic Risk Factors and Food Choices of Adults at Risk for Type 2 Diabetes Mellitus in Cape Town, South Africa
Source: Nutrients. 2022 Aug 4;14(15):3191. doi: 10.3390/nu14153191 (PMC9370540; doi:10.3390/nu14153191)
Supplement: Supplementary file 1 [file nutrients-14-03191-s001.zip › nutrients-1809783-supplementary.pdf]

Table S1. Main reasons preventing eating fruits and vegetables every day per dietary diversity score category.

Main reason preventing eating fruit/vegetables every day	Total (n = 693)	DD score < 5 (n = 488)	DD score ≥ 5 (n = 205)	*p* Value [a]
Fruit				**<0.001**
Cost/Money	295 (42.6)	230 (47.1)	65 (31.7) *	
Health reasons & personal preference	61 (8.8)	50 (10.2)	11 (5.4) *	
Availability & seasonal availability	27 (3.9)	19 (3.9)	8 (3.9)	
Unsure	114 (16.5)	72 (14.8)	42 (20.5)	
Other [b]	32 (4.6)	21 (4.3)	11 (5.4)	
Not applicable	164 (23.7)	96 (19.7)	68 (33.2)	
Vegetables				**0.003**
Cost/Money	198 (28.6)	156 (32.0)	42 (20.5) *	
Health reasons & personal preference	134 (19.3)	94 (19.3)	40 (19.5)	
Availability & seasonal availability	35 (5.1)	30 (6.1)	5 (2.4) *	
Unsure	30 (4.3)	21 (4.3)	9 (4.4)	
Other [c]	17 (2.5)	10 (2.0)	7 (3.4)	
Not applicable	279 (40.3)	177 (36.3)	102 (49.8)	

Data presented as n (%).

DD- Dietary diversity

[a] Chi-square test used for categorical variables. [b] Other reason preventing eating fruit every day -forgetfulness, lack of desire for fruit, lack of appetite, only eat bananas and pears, ignorance, no time, just lazy, don't buy & no reason,[c] Other reason preventing eating vegetables every day - don't cook everyday & no reason.

* Significant difference between DD score categories at p < 0.05 level.

Bold p value - significant at p < 0.05 & < 0.001 level.

Table S2. Food preference fat on meat and poultry, salt in food and use of margarine, butter & fat as spread according to ethnicity according to dietary diversity score category.

Characteristic	Total (n = 693)	DD score < 5 (n = 488)	DD score ≥ 5 (n = 205)	*p* Value [a]
Eat meat with or without fat				0.440
Do not eat meat	31 (4.5)	21 (4.3)	10 (4.9)	
Meat with fat on	320 (46.2)	233 (47.7)	87 (42.4)	
Meat with fat removed	342 (49.4)	234 (48.0)	108 (52.7)	
Eat chicken with or without skin				**0.045**
Do not eat chicken	1 (0.1)	0 (0.0)	1 (0.5)	
Chicken with skin	399 (57.6)	293 (60.0)	106 (51.7) *	
Chicken without skin	293 (42.3)	195 (40.0)	98 (47.8)	
Preference of amount of salt in food				0.710
Very salty	51 (7.4)	36 (7.4)	15 (7.3)	
Lightly salted	590 (85.1)	418 (85.7)	172 (83.9)	
Not salted	52 (7.5)	34 (7.0)	18 (8.8)	
How much margarine, butter or fat is spread on bread, scones, or crackers				0.638
Very thin	94 (13.6)	67 (13.7)	27 (13.2)	
Thin	288 (41.6)	198 (40.6)	90 (43.9)	
Medium	165 (23.8)	113 (23.2)	52 (25.4)	
Thick	97 (14.0)	74 (15.2)	23 (11.2)	
Do not know	49 (7.1)	36 (7.4)	13 (6.3)	

Data presented as n (%).

DD - Dietary diversity

[a] Chi-square test used for categorical variables

* Significant difference between DD score categories at $p < 0.05$ level.

Bold p value - significant at $p < 0.05$ level.

Table S3. Nutritional status and cardiometabolic risk factors of participants according to gender and dietary diversity categories.

Variable	Gender		*p* Value [a]	Male		*p* Value [a]	Female		*p* Value [a]
	**Male (n = 131)**	**Female (n = 562)**		**DD score < 5 (n = 99)**	**DD score ≥ 5 (n = 32)**		**DD score < 5 (n = 389)**	**DD score ≥ 5 (n = 173)**	
BMI	28.6 (26.5 - 32.6)	36.9 (32.6 - 41.5)	**< 0.001**	28.6 (26.6 - 32.7)	28.7 (24.5 - 32.2)	0.824	37.2 (32.9 - 42.2)	36.7 (31.8 - 40.4)	**0.037**
Normal weight (18.5 – 24.9kg/m^2)	21 (16.0)	8 (1.4) *	**< 0.001**	12 (12.1)	9 (28.1)	0.091	7 (1.8)	1 (0.6)	0.224
Overweight (25.0 – 29.9kg/m^2)	62 (47.3)	66 (12.0) *		50 (50.5)	12 (37.5)		41 (10.6)	25 (14.6)	
Obese (≥ 30 kg/m^2)	48 (38.9)	485 (86.7) *		37 (37.4)	11 (34.4)		340 (87.6)	145 (84.8)	
Total	131 (100)	559 (100)		99 (100)	32 (100)		388 (100)	171 (100)	
WHR	0.96 (0.93 - 1.00)	0.91 (0.85 - 0.97)	**< 0.001**	0.96 (0.93 - 0.99)	0.97 (0.91 - 1.01)	0.850	0.91 (0.86 - 0.97)	0.89 (0.84 - 0.95)	0.610
Normal [b]	16 (13.6)	140 (27.2)	**0.002**	12 (13.2)	4 (14.8)	0.828	88 (24.9)	52 (32.2)	0.082
High [c]	102 (86.4)	374 (72.8)		79 (86.8)	23 (85.2)		265 (75.1)	109 (67.7)	
Total	118 (100)	514 (100)		91 (100)	27 (100)		353 (100)	161 (100)	
Glycaemic status	6.0 (5.0 - 7.9)	6.0 (5.0 - 7.4)	0.964	5.8 (5.0 - 7.5)	6.5 (4.7 - 8.8)	0.881	6.0 (4.9 - 7.3)	6.1 (5.1 - 7.6)	0.657
Normoglycemia (FPG ≤ 6 and 2-hour glucose < 7.8 mmol/L)	90 (69.8)	406 (73.7)	0.665	70 (71.4)	20 (64.5)	0.643	288 (75.6)	118 (69.4)	0.281
Prediabetes ((FPG 6.1-7 and 2-hour glucose ≥ 7.8–11.1 mmol/L)	24 (18.6)	90 (16.3)		18 (18.4)	6 (19.4)		59 (15.5)	31 (18.2)	
Diabetes (FPG > 7 and 2-hour glucose > 11.1 mmol/L)	15 (11.6)	55 (10.0)		10 (10.2)	5 (16.1)		34 (8.9)	21 (12.4)	
Total	129 (100)	551 (100)		98 (100)	31 (100)		381 (100)	170 (100)	
TC	5.0 (4.3 - 5.7)	4.9 (4.2 - 5.7)	0.677	5.0 (4.3 - 5.7)	5.3 (4.2 - 5.8)	0.643	4.9 (4.2 - 5.8)	5.0 (4.3 - 5.6)	0.821
Normal (< 5 mmol/L)	64 (49.6)	290 (52.5)	0.550	49 (50.0)	15 (48.4)	0.876	206 (53.9)	84 (49.4)	0.327
Elevated (≥ 5 mmol/L)	65 (50.4)	262 (47.5)		49 (50.0)	16 (51.6)		176 (46.1)	86 (50.6)	
Total	129 (100)	552 (100)		98 (100)	31 (100)		382 (100)	170 (100)	
HDL-C	1.1 (1.0 - 1.3)	1.2 (1.1 - 1.4)	**< 0.001**	1.1 (1.0 - 1.3)	1.0 (1.0 - 1.3)	0.371	1.2 (1.1 - 1.5)	1.2 (1.1 - 1.4)	0.600
Normal (≥ 1.2 mmol/L)	69 (53.5)	203 (36.9) *	**< 0.001**	50 (51.0)	19 (61.3)	0.318	142 (37.4)	61 (35.9)	0.739
Low (< 1.2 mmol/L)	60 (46.5)	347 (63.1) *		48 (49.0)	12 (38.7)		238 (62.6)	109 (64.1)	
Total	129 (100)	550 (100)		98 (100)	31 (100)		380 (69.1)	170 (100)	
LDL-C	3.1 (2.6 - 3.8)	3.1 (2.4 - 3.7)	0.564	3.1 (2.6 - 3.8)	3.1 (2.6 - 3.9)	0.401	3.0 (2.4 - 3.8)	3.1 (2.5 - 3.7)	0.982
Normal (< 3 mmol/L)	58 (45.0)	245 (44.5)	0.932	44 (44.9)	14 (45.2)	0.980	171 (45.0)	74 (43.5)	0.748
Elevated (≥ 3 mmol/L)	71 (55.0)	305 (55.5)		54 (55.1)	17 (54.8)		209 (55.0)	96 (56.5)	
Total	129 (100)	550 (100)		98 (100)	31 (100)		380 (100)	170 (100)	
TG	1.4 (1.1 - 1.8)	1.2 (0.9 - 1.7)	**< 0.001**	1.4 (1.1 - 1.9)	1.3 (1.2 - 1.7)	0.802	1.2 (0.9 - 1.7)	1.2 (0.8 -1.5)	0.671
Normal (≤ 1.5 mmol/L)	72 (55.8)	379 (68.7) *	**0.005**	52 (53.1)	20 (64.5)	0.263	255 (66.8)	124 (72.9)	0.148
Elevated (> 1.5 mmol/L)	57 (44.2)	173 (31.3) *		46 (46.9)	11 (35.5)		127 (33.2)	46 (27.1)	
Total	129 (100)	552 (100)		98 (100)	31 (100)		382 (100)	170 (100)	

Data presented as median (Interquartile range: IQR) or n (%).

DD- Dietary diversity; BMI – Body Mass Index; WHR- Waist -to-Hip ratio; FPG -Fasting plasma glucose; HDL-C- High density lipoprotein cholesterol; LDL-C -Low density lipoprotein cholesterol; TG – triglyceride.

[a] Chi-square test used for categorical variables and spearman correlation for continuous variables [b] Normal WHR: Males ≤ 0.90cm and Females ≤ 0.85 cm, [c] High WHR: Males > 0.90 cm and Females > 0.85 cm

* Significant difference between the gender categories at 0.05 level.

Bold p value - significant at $p < 0.05$ & < 0.001 level.

Table S4. Nutritional status and cardiometabolic risk factors of participants according to ethnicity and dietary diversity categories.

Variable	Ethnicity		*p* Value [a]	Mixed ancestry		*p* Value [a]	Black		*p* Value [a]
	**Mixed ancestry (n = 281)**	**Black (n = 410)**		**DD score < 5 (n = 193)**	**DD score ≥ 5 (n = 88)**		**DD score < 5 (n = 294)**	**DD score ≥ 5 (n = 116)**	
BMI	34.1 (29.9 - 39.4)	36.8 (30.9 - 41.1)	**< 0.001**	34.4 (29.9 - 40.3)	33.7 (30.0 - 37.2)	0.335	36.7 (31.3 - 41.6)	36.8 (30.6 - 40.6)	0.670
Normal weight (18.5 – 24.9kg/m²)	11 (3.9)	18 (4.4)	**0.020**	10 (5.2)	1 (1.1)	0.257	9 (3.1)	9 (7.8)	0.097
Overweight (25.0 – 29.9kg/m²)	60 (21.4)	69 (16.8)		39 (20.2)	21 (23.9)		52 (17.7)	17 (14.7)	
Obese (≥ 30 kg/m²)	210 (74.7)	323 (78.9) *		144 (74.6)	66 (75.9)		233 (79.3)	90 (77.6)	
Total	281 (100)	410 (100)		193 (100)	87 (100)		294 (100)	116 (100)	
WHR	0.94 (0.89 - 0.98)	0.91 (0.84 - 0.97)	**< 0.001**	0.94 (0.89 - 0.99)	0.92 (0.87 - 0.97)	0.282	0.91 (0.85 - 0.97)	0.89 (0.84 - 0.97)	0.224
Normal [b]	37 (13.9)	119 (32.5)	**< 0.001**	23 (12.6)	14 (16.9)	0.348	77 (29.5)	42 (40.0)	0.052
High [c]	229 (86.1)	247 (67.5)		160 (87.4)	69 (83.1)		184 (70.5)	63 (60.0)	
Total	266 (100)	366 (100)		183 (100)	83 (100)		261 (100)	105 (100)	
Glycaemic status	6.4 (5.2 - 8.3)	5.8 (4.9 -7.0)	**< 0.001**	6.2 (5.1 - 8.4)	6.7 (5.6 - 8.4)	0.480	5.8 (4.9 - 7.0)	5.8 (4.6 - 7.1)	0.771
Normoglycemia (FPG ≤ 6 and 2-hour glucose < 7.8 mmol/L)	178 (64.5)	317 (78.9)	**< 0.001**	128 (67.4)	50 (58.1)	0.291	230 (79.9)	87 (76.3)	0.163
Prediabetes ((FPG 6.1-7 and 2-hour glucose ≥ 7.8–11.1 mmol/L)	65 (23.6)	49 (12.2) *		40 (21.1)	25 (29.1)		37 (12.8)	12 (10.5)	
Diabetes (FPG > 7 and 2-hour glucose > 11.1 mmol/L)	33 (12.0)	36 (9.0)		22 (11.6)	11 (12.8)		21 (7.3)	15 (13.2)	
Total	276 (100)	402 (100)		190 (100)	86 (100)		288 (100)	114 (100)	
TC	5.4 (4.6 - 6.1)	4.7 (4.0 - 5.5)	**< 0.001**	5.3 (4.5 - 6.3)	5.4 (4.7 - 5.9)	0.977	4.7 (4.1 - 5.5)	4.7 (3.8 - 5.3)	0.662
Normal (< 5 mmol/L)	111 (39.9)	243 (60.6)	**< 0.001**	81 (42.2)	30 (34.9)	0.250	174 (60.6)	69 (60.5)	0.985
Elevated (≥ 5 mmol/L)	167 (60.1)	158 (39.4)		111 (57.8)	56 (65.1)		113 (39.4)	45 (39.5)	
Total	278 (100)	401 (100)		192 (100)	86 (100)		287 (100)	114 (100)	
HDL-C	1.2 (1.1 - 1.4)	1.2 (1.1 - 1.4)	0.172	1.2 (1.1 - 1.4)	1.2 (1.1 - 1.3)	0.973	1.2 (1.1 - 1.4)	1.3 (1.1 - 1.4)	0.453
Normal (≥ 1.2 mmol/L)	123 (44.2)	148 (37.1)	0.062	84 (43.8)	39 (45.3)	0.804	107 (37.5)	41 (36.0)	0.768
Low (< 1.2 mmol/L)	155 (55.8)	251 (62.9)		108 (56.3)	47 (54.7)		178 (62.5)	73 (64.0)	
Total	278 (100)	399 (100)		192 (100)	86 (100)		285 (100)	114 (100)	
LDL-C	3.4 (2.8 - 4.2)	2.8 (2.3 - 3.4)	**< 0.001**	3.4 (2.8 - 4.3)	3.6 (2.9 - 3.9)	0.921	2.8 (2.3 - 3.5)	2.8 (2.1 - 3.3)	0.592
Normal (< 3 mmol/L)	81 (29.1)	222 (55.6)	**< 0.001**	59 (30.7)	22 (25.6)	0.383	156 (54.7)	66 (57.9)	0.566
Elevated (≥ 3 mmol/L)	197 (70.9)	177 (44.4)		133 (69.3)	64 (74.4)		129 (45.3)	48 (42.1)	
Total	278 (100)	399 (100)		192 (100)	86 (100)		285 (100)	114 (100)	
TG	1.4 (1.1 - 1.9)	1.1 (0.8 - 1.5)	**< 0.001**	1.4 (1.1 - 1.9)	1.3 (1.0 - 1.8)	0.551	1.1 (0.9 - 1.6)	1.1 (0.8 - 1.4)	0.341
Normal (≤ 1.5 mmol/L)	158 (56.8)	293 (73.1) *	**< 0.001**	104 (54.2)	54 (62.8)	0.180	203 (70.7)	90 (78.9)	0.094
Elevated (> 1.5 mmol/L)	120 (43.2)	108 (26.9) *		88 (45.8)	32 (37.2)		84 (29.3)	24 (21.1)	
Total	278 (40.9)	401 (59.1)		192 (100)	86 (100)		287 (100)	114 (100)	

Data presented as median (Interquartile range: IQR) or n (%).
DD- Dietary diversity; BMI – Body Mass Index; WHR- Waist -to-Hip ratio; FPG -Fasting plasma glucose; HDL-C- High density lipoprotein cholesterol; LDL-C -Low density lipoprotein cholesterol; TG – triglyceride.
[a] Chi-square test used for categorical variables and spearman correlation for continuous variables. [b] Normal WHR: Males ≤ 0.90cm & Females ≤ 0.85 cm, [c] High WHR: Males > 0.90cm & Females > 0.85 cm.
* Significant difference between the ethnicity categories at 0.05 level.
Bold p value - significant at $p < 0.05$ & < 0.001 level.
